# DEFENSIVENESS AGAINST INTERGROUP BIASES ACROSS ADULTHOOD

**DOI:** 10.1093/geroni/igad104.3797

**Published:** 2023-12-21

**Authors:** Jennifer Beatty, Patrick Hill

**Affiliations:** Washington University in St. Louis, St. Louis, Missouri, United States; Washington University in St. Louis, St. Louis, Missouri, United States

## Abstract

Research has demonstrated the pernicious role of discrimination against marginalized groups. Less work has considered the perspective of the transgressor and how defensive they may be when told they were biased. Such work is critical for understanding which individuals may be more willing to acknowledge and integrate feedback about their personal biases, allowing them to avoid future discriminatory and biased actions. Moreover, older adults may be more defensive in the face of feedback if they are less willing to change well-entrenched personal attitudes. The current study developed a new measure of feedback defensiveness and investigated whether defensiveness levels increase or decrease with age. In a sample of U.S. White adults (n = 303; age range: 19 to 85), we found that individuals reported being more defensive in the face of negative feedback about intergroup biases relative to feedback about other aspects of the self. Correlational analyses found that these biases were weaker among individuals who reported a stronger sense of personal identity. Critically, defensiveness against intergroup biases was initially unassociated with age, but when we consider individuals who avoid identity information, there is a modest age effect. This data challenges the assumption that all older adults would be defensive to feedback about their bias and considers the role of one’s sense of identity. These findings have clear implications for how clinicians, practitioners, and researchers should focus their efforts to reduce discriminatory events by alerting them to which individuals may be more willing to accept feedback about their biases.

